# Predictive Habitat Modelling as a Tool to Assess the Change in Distribution and Extent of an OSPAR Priority Habitat under an Increased Ocean Temperature Scenario: Consequences for Marine Protected Area Networks and Management

**DOI:** 10.1371/journal.pone.0068263

**Published:** 2013-07-22

**Authors:** Kate S. G. Gormley, Joanne S. Porter, Michael C. Bell, Angela D. Hull, William G. Sanderson

**Affiliations:** 1 Centre for Marine Biodiversity and Biotechnology, School of Life Sciences, Heriot Watt University, Riccarton, Edinburgh, United Kingdom; 2 Institute for Building and Urban Design, School of the Built Environment, Heriot Watt University, Riccarton, Edinburgh, United Kingdom; 3 International Centre for Island Technology, Heriot Watt Institute of Petroleum Engineering, the Old Academy, Stromness, Orkney, United Kingdom; Institute of Marine Research, Norway

## Abstract

The aims of this study were to determine the extent and distribution of an OSPAR priority habitat under current baseline ocean temperatures; to illustrate the prospect for habitat loss under a changing ocean temperature scenario; and to demonstrate the potential application of predictive habitat mapping in “future-proofing” conservation and biodiversity management.

Maxent modelling and GIS environmental envelope analysis of the biogenic bed forming species, *Modiolus modiolus* was carried out. The Maxent model was tested and validated using 75%/25% training/test occurrence records and validated against two sampling biases (the whole study area and a 20km buffer). The model was compared to the envelope analysis and the area under the receiver operating characteristic curve (Area Under the curve; AUC) was evaluated.

The performance of the Maxent model was rated as ‘good’ to ‘excellent’ on all replicated runs and low variation in the runs was recorded from the AUC values. The extent of “most suitable”, “less suitable” and “unsuitable” habitat was calculated for the baseline year (2009) and the projected increased ocean temperature scenarios (2030, 2050, 2080 and 2100). A loss of 100% of “most suitable” habitat was reported by 2080.

Maintaining a suitable level of protection of marine habitats/species of conservation importance may require management of the decline and migration rather than maintenance of present extent. Methods applied in this study provide the initial application of a plausible “conservation management tool”.

## Introduction

It is widely accepted that the natural distribution patterns of organisms are primarily driven by their environmental requirements [1]; and that climate change is potentially having an impact on natural distribution patterns through range expansion, contraction and migration [2,3]. The effect which climate change has on geographic distribution is often assessed in terms of potential envelopes/spatial niches shifting in altitude, longitude or latitude; and this influence could, in turn, threaten biodiversity and the conservation of many species [3-5].

Priority marine habitats (determined as ‘threatened and/or declining species and habitats’ under the OSPAR Convention for the Protection of the Marine Environment of the north-east Atlantic 1992) are considered to be of greatest marine nature conservation importance within the North-East Atlantic and are being used to prioritise marine biodiversity conservation and protection under Annex V of the OSPAR Convention 1992. The maintenance of priority habitats will also contribute to the achievement of ‘Good Environmental Status’ (GES) under the European Union (EU) Marine Strategy Framework Directive (MSFD; 2008/56/EC; see also 6). Appropriate area-based management strategies, including a network of Marine Protected Areas (MPAs), are being considered under the MSFD with these and other habitats in mind [7].

Data on the distribution of marine species and habitats are often limited, mainly because of the complexity and costs of surveying and sampling extensive sea areas. For example, habitat maps based on survey data and ground truthing currently cover just 10% of the UK continental shelf [8]. The use of predictive species distribution modelling might therefore provide a suitable tool to fill knowledge gaps, but it may be subject to the issue of over-prediction of range when studying individual species [9]. Ross and Howell [9] acknowledged that a more robust approach might be to apply predictive modelling methods to a habitat formed by a species, rather than to the indicator species itself. This principle has been adopted in the present study.

The objective of this study is to explore the use of a predictive Species Distribution Model (SDM) and a Geographical Information System (GIS) based Environmental Envelope Analysis (EEA) method to create modelled habitat maps for a priority habitat: the biogenic horse mussel reefs formed by the bivalve mollusc *Modiolus modiolus* (Linnaeus, 1758).

Although *M* *. modiolus* is a widespread and common species, actual horse mussel beds are limited in their distribution [10] and often represent biodiversity ‘hotspots’ e.g. [11], some of which have been, or are in the process of being selected for Marine Protected Area status [12-14]. *M* *. modiolus* is an Arctic-Boreal species, with a distribution range covering the seas around Scandinavia (including Skagerrak and Kattegat) and Iceland south towards the Bay of Biscay [15-17]. *M* *. modiolus* is known to inhabit the subtidal and lower intertidal region of the northern Atlantic and Pacific oceans [15], often in water depths between 5 and 50m; however some individuals have been found at a depth of 280m [15,18]. Dense aggregations/beds reach their southerly limit around the British Isles, in the Irish Sea. This suggests that their occurrence around the British Isles may be vulnerable to a long-term rise in water temperature [16,18].

*M* *. modiolus* beds are thought to play an important role in benthic productivity and seabed stabilisation. The beds contribute to high biodiversity and may provide refugia and feeding opportunities to other marine organisms [10,19,20]. Although maps of bed distribution have been created, there is still a considerable amount of uncertainty as to the true extent of these beds within the OSPAR region [21].

The primary goal of this study is to use publicly available datasets to test the modelling approaches for a *M* *. modiolus* habitat case example, to see whether it may provide a new tool to inform the MSFD spatial management process for key habitats. The models will be applied to determine the extent of habitat suitable for *M* *. modiolus* beds under current baseline conditions; predict habitat loss under an increased ocean temperature scenario; and demonstrate the application of a predictive habitat mapping tool for “future-proofing” spatial planning for habitats and biodiversity management planning.

## Methods

### *Modiolus modiolus* Occurrence Data

**Figure 1:**
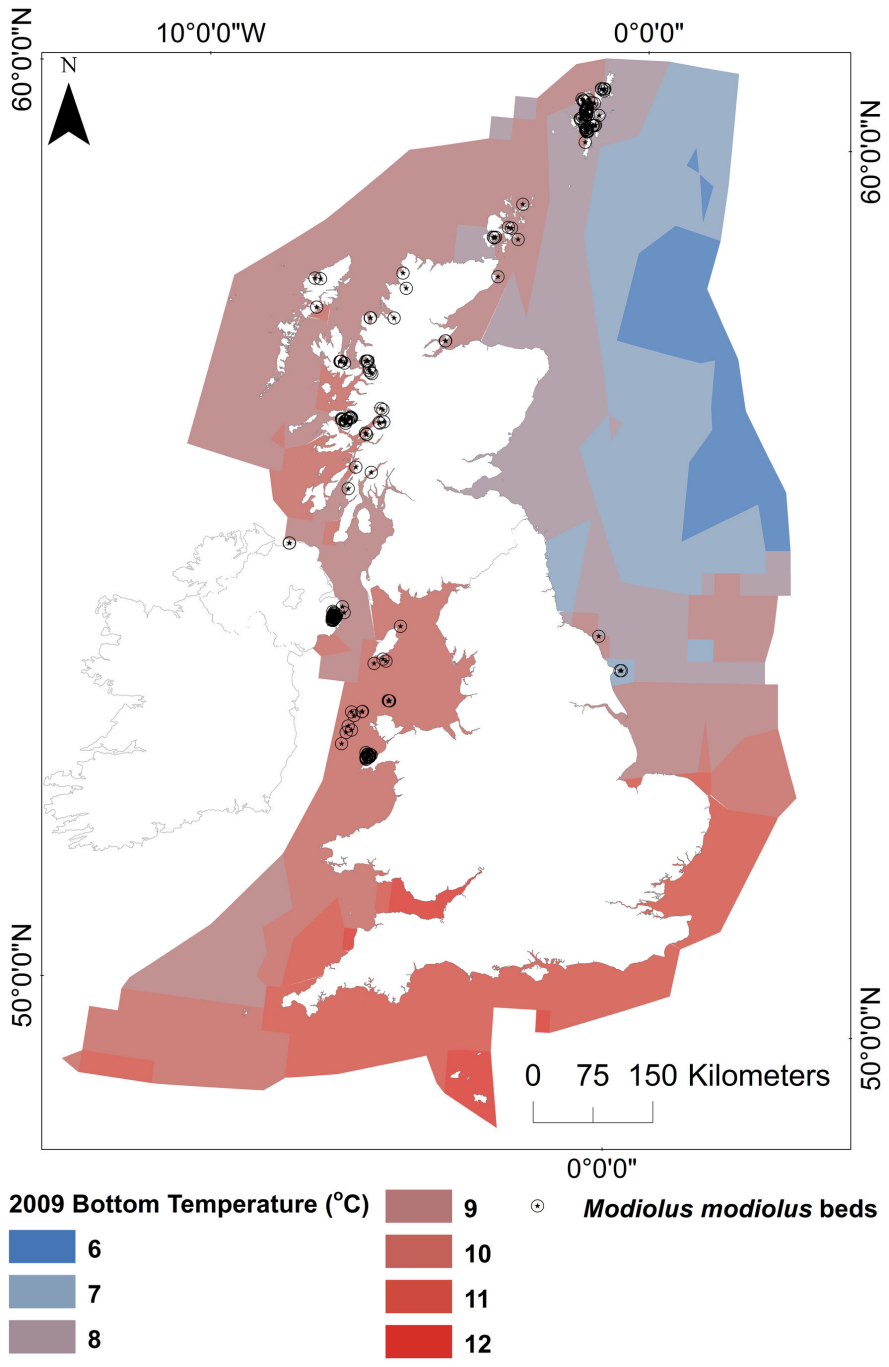
Study area, current known distribution of *Modiolus modiolus* (Linnaeus, 1758) beds and illustrated baseline (2009) seabed temperature ( ^o^C). Projection: WGS 1984 UTM 31N.

The *M* *. modiolus* bed occurrence records were extracted from the 2011 OSPAR priority habitats dataset [22] and corrected based on areas of uncertainty published by Rees [21]. The data were supplemented with occurrence records collected during more recent UK surveys [13,14,23]. A total of 215 occurrence records were obtained (Figure 1). As a result of the limited geographical coverage of some of the environmental layers, 82 records were excluded because they did not coincide with the environmental layers.

### Environmental Data

Data on environmental variables of potential biological relevance to *M* *. modiolus* were obtained from publically available sources (Table 1) then assigned to a 0.005^o^ grid using ArcMap 9.3 Geographical Information System (GIS) software. Temperature, depth, substratum, water movement and salinity were chosen based on the *M* *. modiolus* environmental requirements as outlined by Holt et al. [18], but water quality and suspended sediment were not available for inclusion in this model.

**Table 1:** Environmental variables and data sources.

**Variable**	**Source**
Bathymetry: depth (m)	GEBCO_08 30-second arc Bathymetry resolution [63]
Slope: percentage gradient of the seafloor (%)	Adapted in ArcGIS 9.3 from: GEBCO_08 30-second arc Bathymetry resolution [63]
Sea Bottom Temperature: climatological annual mean sea bottom temperature (^o^C). Adapted from NOAA depth interval data	NOAA, World Ocean Atlas [24]
Bottom Salinity: climatological annual mean sea bottom salinity (PSS). Adapted from NOAA depth interval data	NOAA, World Ocean Atlas [64]
Landscape: seabed landscape features [Broad patterns in seabed character, such as seabed morphology determined by major geological and hydrographic processes]	UKSeaMap/MESH webGIS [65] http://www.searchmesh.net/ (“Marine Landscapes” layer on interactive map)
Current Speed: average spring current speed (ms^-1^)	Atlas of UK marine renewable energy resources [66] Supplemented by: Current speed data on UKHO Navigation Charts [67] and BODC oceanographic data [68]

### Increased Ocean Temperature Scenario

Increased ocean temperature scenarios were established for the following epochs: 2009 (Figure 1), 2030, 2050, 2080 and 2100 based on Locarnini et al. [24] and the International Panel on Climate Change (IPCC) scenario planning methodology [25]. Predictions were based on the IPCC climate change scenario A1B in which a 4^o^C increase in ocean surface temperature would occur by 2100 [25]. A linear increase in ocean bottom temperature was calculated between 2009 and 2100, therefore increases of 0.92^o^C, 1.80^o^C and 3.12^o^C were expected for 2030, 2050 and 2080 respectively.

Model scenarios assumed a uniform increase in temperature over the entire spatial domain and throughout the water column.

### Environmental Envelope Analysis

Initial baseline species distribution analysis was carried out through the creation of an environmental envelope for *M* *. modiolus* bed populations in ArcMap 9.3. The *M* *. modiolus* bed occurrence records were grouped into populations based on their location and proximity to each other. Populations were selected if the occurrence records were within 10km of each other, excluding areas of obvious boundaries, e.g. land or sealochs etc. Within this 10km population grouping, the individual occurrence records were given a 1km buffer which would represent bed extent within that particular population. Environmental layers were plotted in vector format and overlaid with the population records. The "preferred range" of environmental attributes was characterised in terms of the interquartile ranges of the environmental variable values over the occurrence locations.

The "preferred range" for the landscape was calculated based on qualitative data (therefore the interquartile range calculation within ArcMap was not suitable). The area of overlap for each population and landscape type was calculated. The percentage of each landscape type inhabited by a population was calculated (landscape range), ranked, then the median and maximum of these percentages (landscape range) was determined. The "preferred" landscape types were determined as representing those that were inhabited by the majority of the populations (≥ the median of the landscape range).

Areas where "preferred range" attributes occurred for all overlying environmental layers were classed as the environmental envelope for horse mussel beds.

### Species Distribution Model

Maxent is a predictive method that models the geographic distribution of species using presence-only data. Probability of occurrence is modelled in relation to environmental variables under the assumption that the species distribution will follow the property of maximum entropy [26-28]. Maxent has been used in a number of comparative studies examining the effectiveness of species distribution modelling (SDM) in the marine environment [28-30] and is considered to be reliable in this context [29].

### Model Validation

The model predictions were tested using the ‘Area Under the Curve’ (AUC) produced by Maxent. The area under the Receiver Operating Characteristic (ROC) curve is a widely used test statistic which measures model performance [28]. The AUC varies between 0 and 1, with values above 0.9 indicating excellent prediction, between 0.7 and 0.9 indicating good prediction, below 0.7 indicating poor prediction, and below 0.5 no better than random [29].

Owing to the lack of independent test datasets, models were assessed by 2-fold cross validation on ten replicate runs [28]. The occurrence dataset was randomly split in ArcMap 9.3 using the Hawths Analysis Tools for ArcGIS extension [31] each containing a randomly selected 75% of records for model training and the corresponding 25% for model testing. A further model cross-validation was run using the full occurrence dataset randomly split into a 90% training/10% test dataset internally using the Maxent random test setting.

No absence data were available and therefore 10,000 randomly chosen pseudo-absence/background points were run. Selecting the background points from the whole study area may artificially inflate the AUC value, especially if the geographic area is particularly large or the area of suitable habitat is small in relation to the whole study area [28]. During model evaluation, models were tested using background points selected from within a 20km buffer of the known occurrence locations (bias model) and compared with models run with background points selected from the whole study area (global model).

It was considered that the landscape layer might artificially influence the distribution of suitable habitat within the model, therefore, jack-knife contributions of each variable were measured to test the contribution of each variable to the model.

The tested models were visually inspected and compared to the environmental envelope analysis, and occurrence data. This enabled the assessment of model plausibility with respect to the known distribution and areas of suitable habitat outside known occurrence range (over-prediction) [28].

### Probability of Habitat Distribution

The probability of occurrence values (0 to 1) estimated in the Maxent model training and projection runs were separated into 10 bands and the area (Km^2^) covered by each band was calculated.

The 10 probability bands were further separated into 3 categories for MPA region assessment:

i) 0.5–1.0 representing a situation where *M* *. modiolus* beds may be more likely to occur (“most suitable habitat”);
ii) 0.1-0.49 representing a situation where *M* *. modiolus* beds are less likely to occur (“less suitable habitat”); and
iii) 0.0–0.09 representing a situation where *M* *. modiolus* beds are highly likely not to occur (“unsuitable”).

In this study, MPA Regions are defined as designated regions of search for potential MPAs within UK waters (200nm limit).

## Results

### Environmental Envelope Analysis

The environmental envelope analysis method was applied to the *M* *. modiolus* bed population locations (Figure 1) and is a simple summarisation of potential suitable habitat for *M* *. modiolus* beds within UK waters. Table 2 outlines the environmental envelope calculated for *M* *. modiolus* beds.

**Table 2:** The selected Environmental Envelope for *Modiolus modiolus* (Linnaeus, 1758) beds.

**Environmental Layer**	**Preferred Range**
Temperature:	9 to 10 ^o^C
Landscape:	Sealoch
	Shallow coarse sediment plain - moderate tide stress
	Shallow coarse sediment plain - weak tide stress
	Shelf coarse sediment plain - moderate tide stress
	Shallow sand plain
	Embayment
	Shallow mixed sediment plain - weak tide stress
	Shallow mud plain
	Shelf coarse sediment plain - strong tide stress
	Photic rock
Bathymetry:	-20 to 0 m
Current Speed:	0.5 to 1.115 m/s
Slope:	0 to 0.345%
Salinity:	34 to 35 ppt

**Figure 2:**
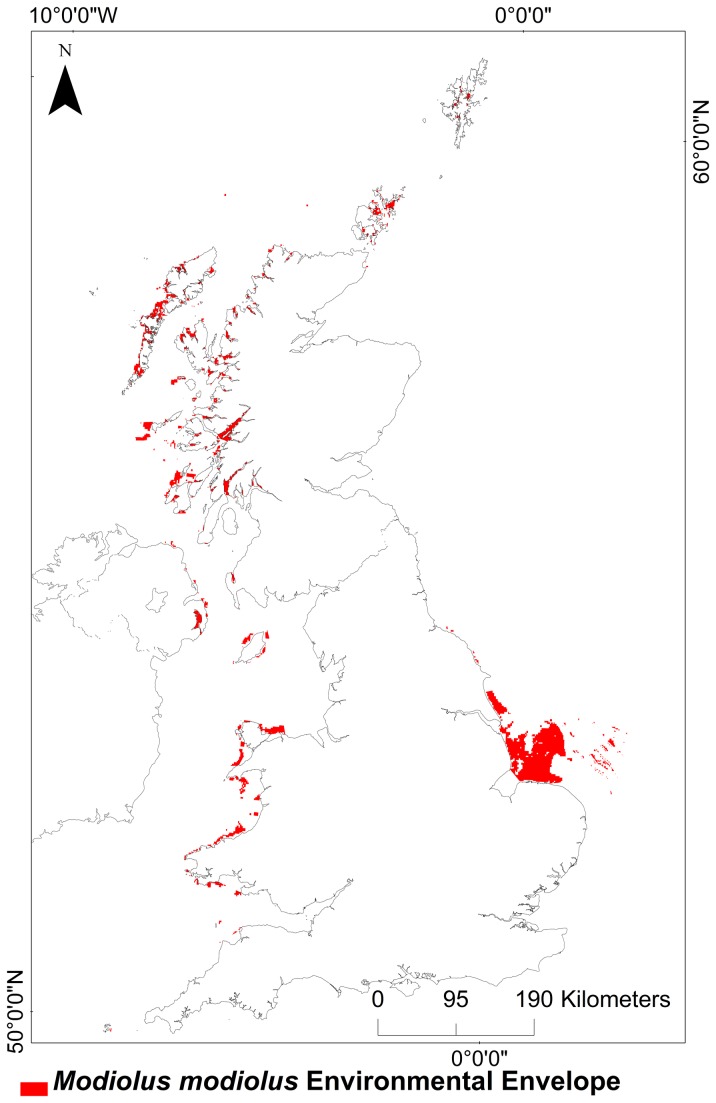
ArcMap calculated Environmental Envelope for *Modiolus modiolus* (Linnaeus, 1758) beds. Projection: WGS 1984 UTM 31N.

Figure 2 illustrates the environmental envelope for *M* *. modiolus* beds and represents areas of suitable *M* *. modiolus* bed habitat generated by the envelope analysis.

This method indicates that the west of Scotland, Strangford Lough in Northern Ireland, Wales, Orkney and Shetland are the most suitable areas for *M* *. modiolus* beds, with more scattered areas around the Isle of Man and the east coast of England.

When the envelope analysis was applied to the projected climate change scenarios, results indicated that there would be a decrease of potentially suitable habitat by 2050 (58% loss by 2030; and 98% loss by 2050) and complete loss of suitable *M* *. modiolus* bed habitat by 2080.

The envelope analysis was re-run for the baseline model, excluding the landscape environmental layer (to test for environmental variable bias) and a small increase in suitable habitat was noted, however, results still showed the same distribution pattern as before, with a slight increased presence around the coast of Wales, east England and south west Scotland. This comparison shows that the landscape layer did not have a disproportionate effect on the baseline model outcome.

### Species Distribution Model

#### Model Selection

The Maxent model was trained using cross-validation of 2 externally selected sub-sets of the 2009 baseline data and further trained for an internally selected sub-set within Maxent’s automated validation test. The training AUC values, shown in Table 3, ranged from 0.92 to 0.99 with little variation shown over the 10 replicates (maximum difference from 0 to 0.006). The test AUC values ranged from 0.86 to 0.98 and showed slightly higher variation over the replicated runs (maximum difference 0.008 to 0.047).

**Table 3:** Threshold-independent area under the curve (AUC) indices for *Modiolus modiolus* (Linnaeus, 1758) habitat model.

	**Average AUC Test Statistic**			
	**Training**		**Testing**	
**Model (Training/Test)**	**Bias**	**Global**	**Bias**	**Global**
**Set 1 (75/25%)**	0.92 ±0.003	0.98 ±0.001	0.86 ±0.051	0.97 ±0.023
**Set 2 (75/25%)**	0.94 ±0.003	0.99 ±0.001	0.90 ±0.047	0.97 ±0.043
**All (90/10%)**	0.93 ±0.006	0.99 ±0.000	0.92 ±0.039	0.98 ±0.008
**Final model (90/10%)**	**0.93**	**0.99**	**0.88**	**0.97**

Test statistic values decreased when calculated using pseudo-absences restricted to 20 km from occurrence records.

A final model was run for each of the sampling scenarios using the full occurrence records and a 90%/10% training/test ratio run on a single replicate. The AUC values for the final model ranged from 0.93 to 0.99 for model training and 0.88 to 0.97 from model testing and generally equalled the average of the cross-validated models, indicating little variation between the overall model test statistics. Overall, the environmental variable with the highest gain when used in isolation was landscape, which therefore appears to have the most useful information by itself when determining the location of suitable habitat. In contrast, when Bathymetry was omitted the jack knife analysis showed the lowest gain, indicating that the Bathymetry variable has the most information not present in the other variables, when determining location of suitable habitat. The AUC values remained above 0.96 for each model run following omission of each environmental variable in turn; this indicates ‘excellent’ model performance.

**Figure 3:**
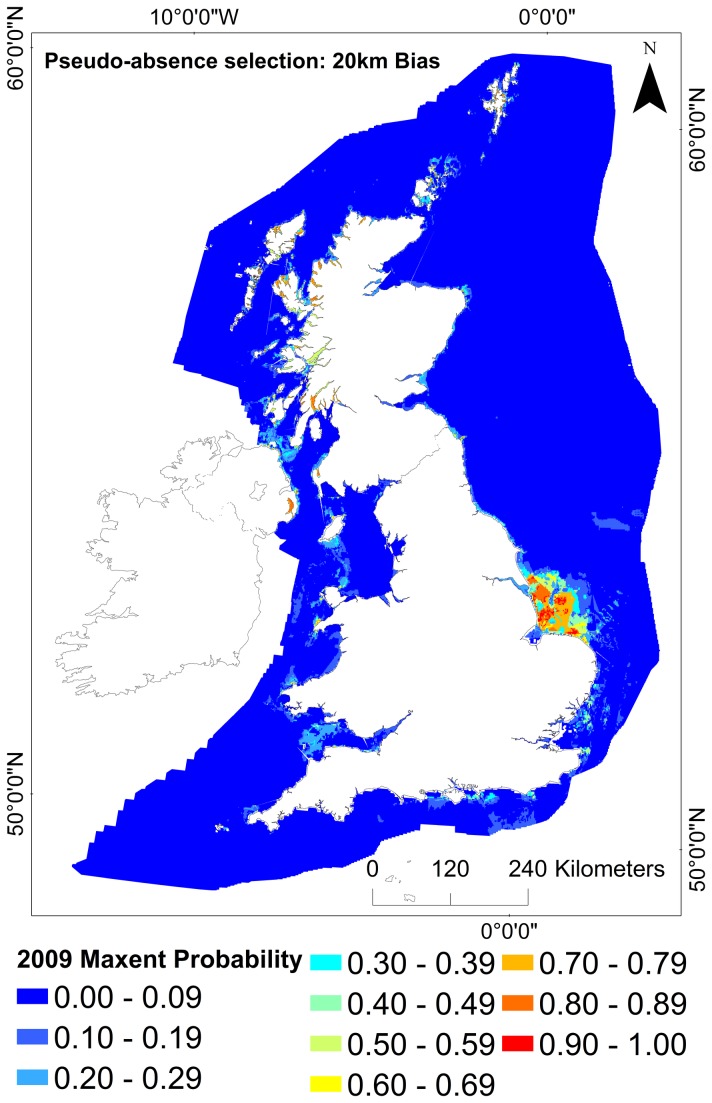
Full model prediction map (Maxent output) for *Modiolus modiolus* (Linnaeus, 1758) beds under baseline conditions (2009). Model sampling bias: 20km. Projection: WGS 1984 UTM 31N.

**Figure 4:**
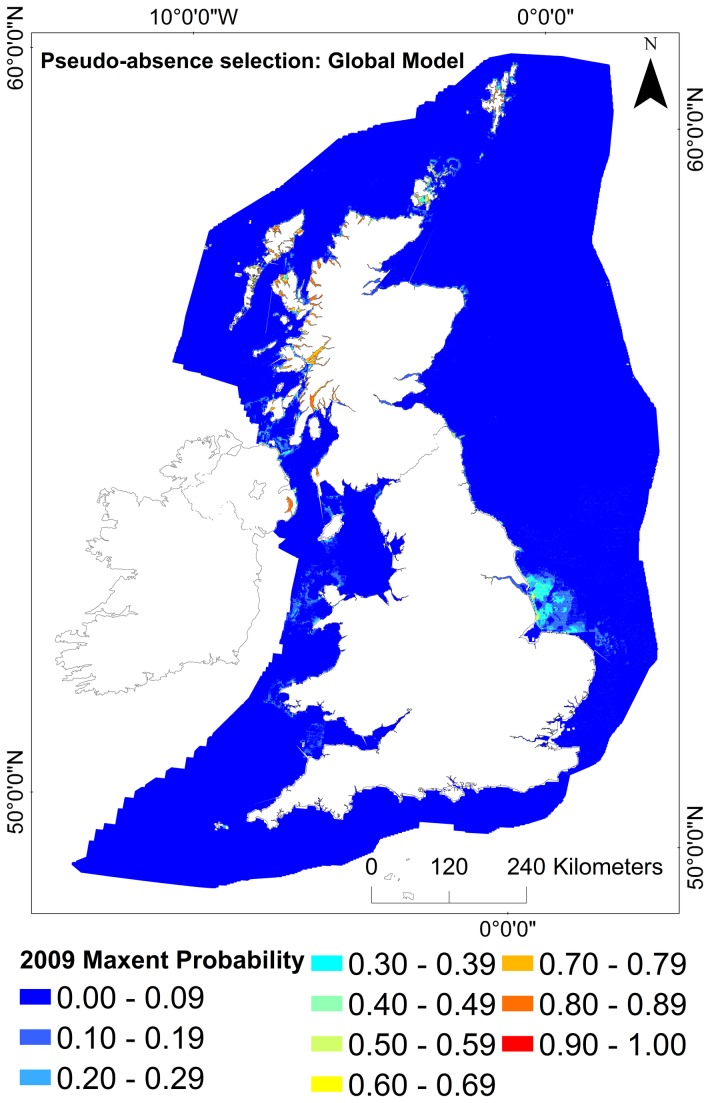
Full model prediction maps (Maxent output) for *Modiolus modiolus* (Linnaeus, 1758) beds under baseline conditions (2009). Model sampling bias: Global. Projection: WGS 1984 UTM 31N.

Pseudo-absence selection models were compared. The models where pseudo-absences were chosen from within 20km of the known occurrence records predicted suitable habitat to occur to the west of Scotland and Northern Ireland, but with the highest probability of suitable habitat occurring on the North Norfolk sandbanks. There were also areas of low probability predicted in the English Channel and a lack of suitable habitat predicted around Orkney (Figure 3). In comparison, in the models where pseudo-absences were selected from the whole study area, the highest probability of suitable habitat occurring was observed predominantly to the West of Scotland, Shetland and Northern Ireland. The area around the Norfolk sandbanks showed lower levels of probability (Figure 4).

The “most suitable” habitat output (probability ≥0.5) for 2009 from Maxent were compared with the environmental envelope.

The output of the Environmental Envelope Analysis (EEA) showed a 50% overlap of the "most suitable" habitat predicted by the Maxent global sampling model; with an overlap of 22% of the "less suitable" habitat (Table 4) and <1% of the “unsuitable” habitat.

**Table 4:** Comparison of Environmental Envelope Analysis and Maxent model outputs. Overlap area calculations.

**Method/Model**		**Area (Km** ^2^ **)**	**Percentage of Maxent overlapped by envelope**	**Combined overlap (excluding "unsuitable" habitat)**	**Percentage "over prediction" (model vs envelope)**
**Envelope Analysis**		7,009	n/a	n/a	n/a
**Global model**	**"Most Suitable"**	2,191	50%	26%	58%
	**"Less Suitable"**	14,390	22%	26%	58%
**Bias model**	**"Most Suitable"**	6,471	55%	16%	81%
	**"Less Suitable"**	29,659	8%	16%	81%

#### Model Projections

**Figure 5:**
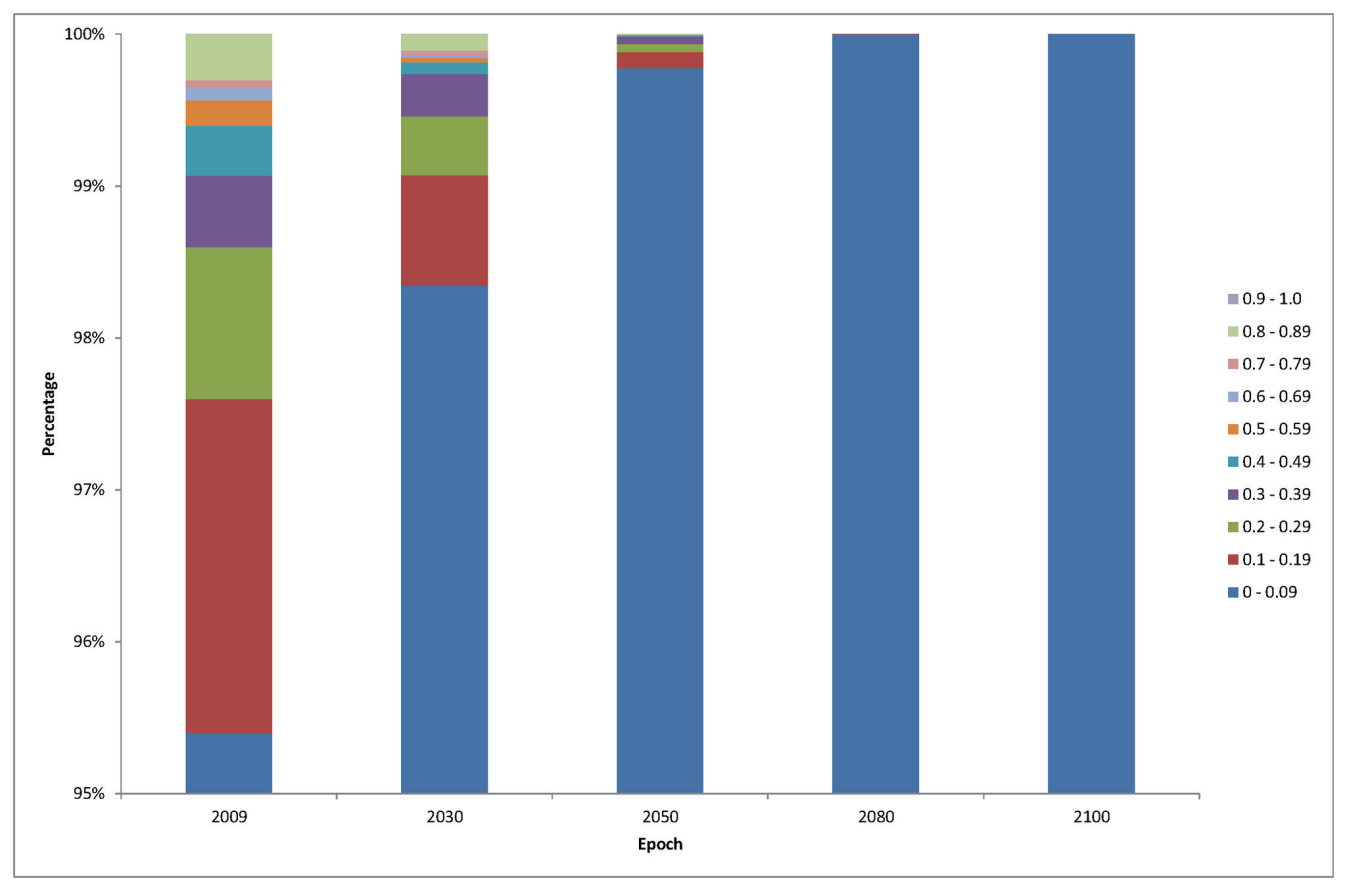
Percentage of area suitable for *Modiolus modiolus* (Linnaeus, 1758) habitat based on different probability scenarios.

**Figure 6:**
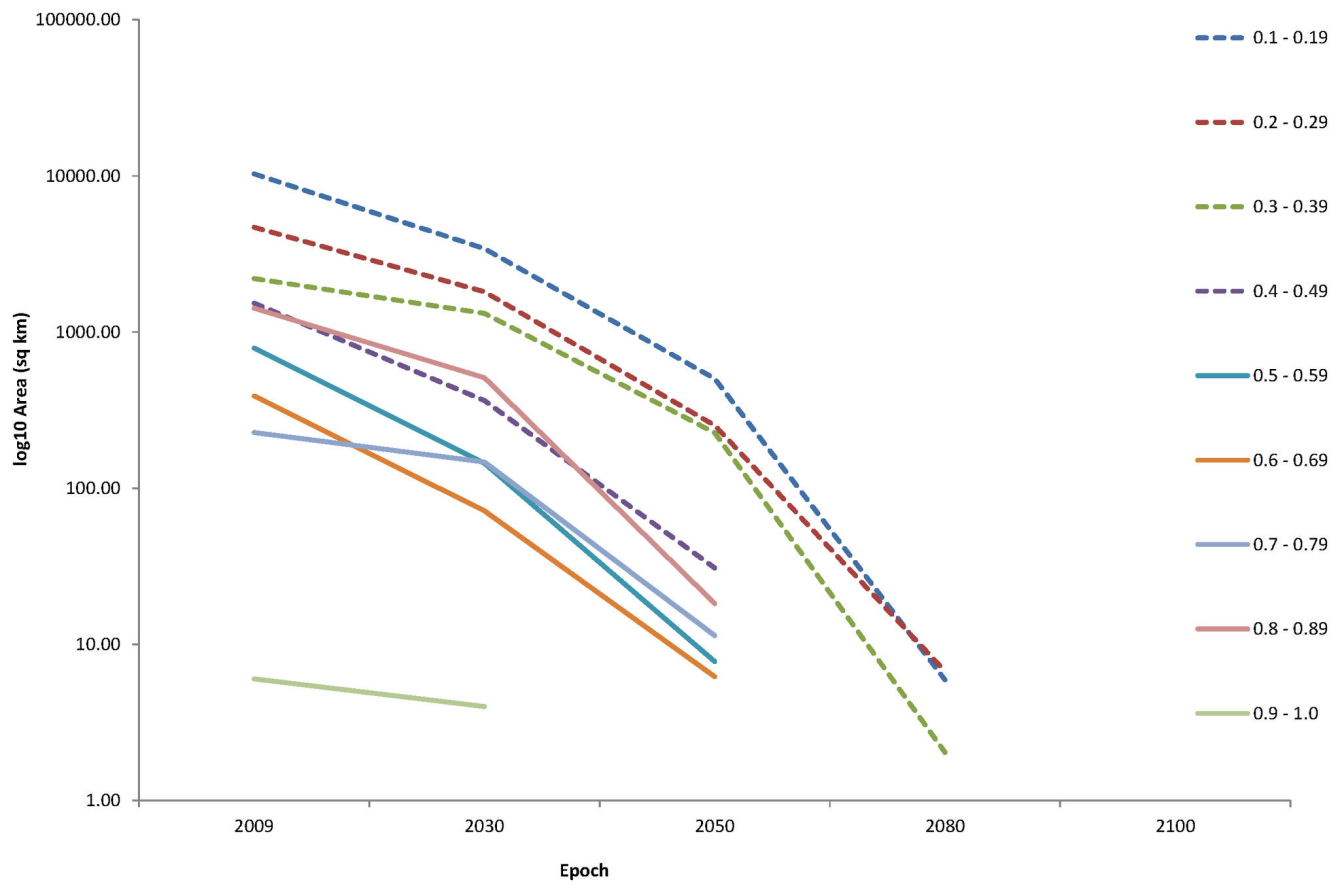
Change in suitable *Modiolus modiolus* (Linnaeus, 1758) habitat occurrence area (Km^2^) (Log_10_) between 2009 and 2100. Illustration of speed of habitat loss.

**Figure 7:**
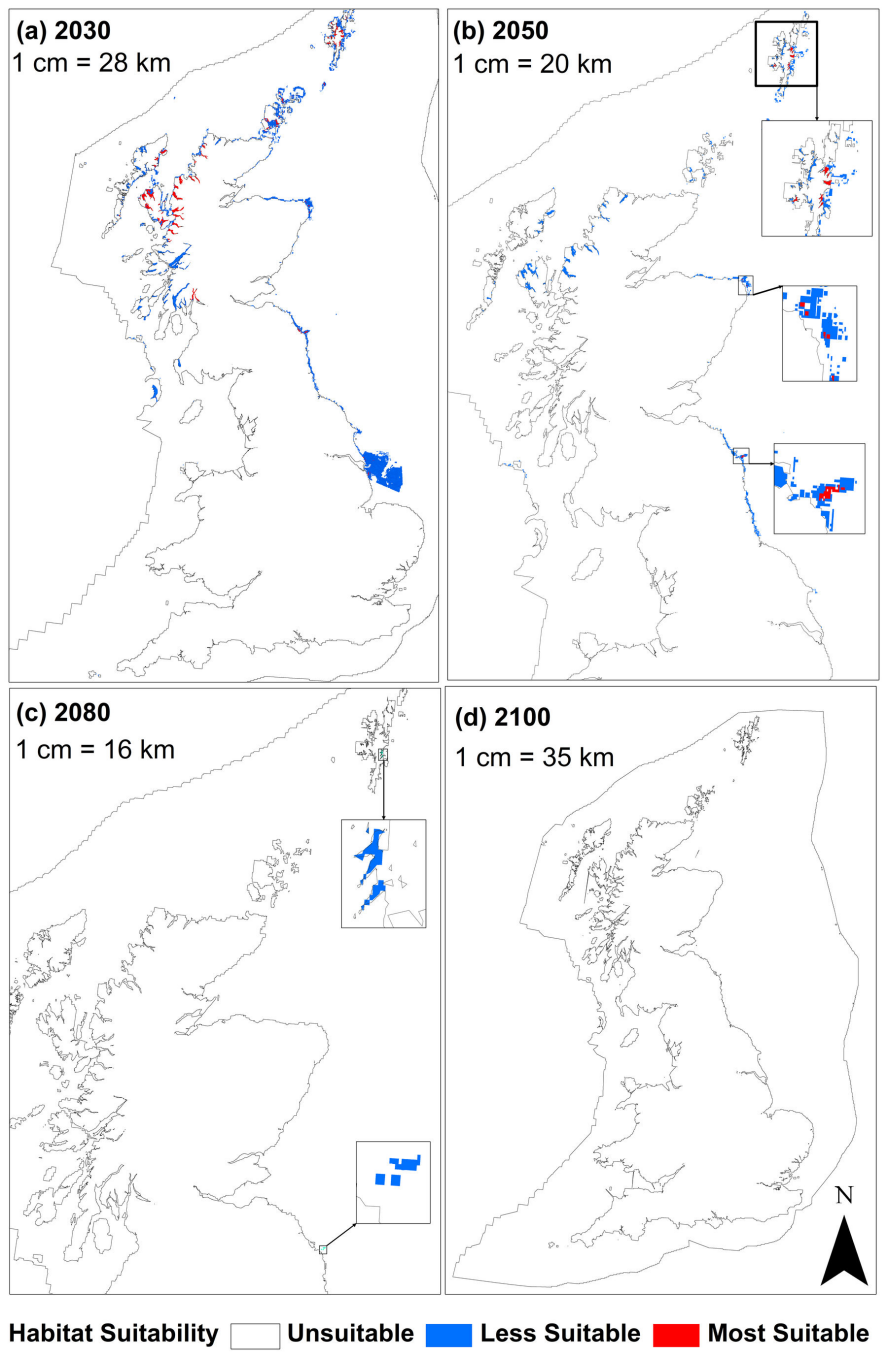
Full model prediction maps for *Modiolus modiolus* (Linnaeus, 1758) beds for the 4 projected climate change epochs (a) 2030, (b) 2050, (c) 2080 and (d) 2100. Projection: WGS 1984 UTM 31N.

The selected baseline model was projected against the predicted 2030, 2050, 2080 and 2100 conditions. Figure 5 illustrates that the percentage of sea area suitable for *M* *. modiolus* beds decreases rapidly over the 4 projected epochs with a 100% loss of *M* *. modiolus* bed habitat predicted by 2100. The 10 probability bands were separated into 3 categories for ease of examination and discussion: “most suitable” (MS), “less suitable” (LS) and “unsuitable” (US) habitat. Calculated areas indicated a 100% loss of “most suitable” habitat by 2080 (Figure 5). Figure 6 illustrates the rapidity of habitat loss of the epochs. The steepest decline of potential habitat occurs in bands 0.1 to 0.39 between 2050 and 2080, and band 0.8 to 0.89 between 2030 and 2050. The modelled projections are illustrated in Figure 7. The extent of predicted distribution as represented by the shading, decreases significantly over the 4 epochs.

### MPA Region Assessment

The area of MS, LS and US habitat within each MPA region was calculated over the 5 epochs and these data are summarised in Table 5. The results show that there are some MPA regions that are potentially more important to *M* *. modiolus* beds than others. The area and percentage loss of “most suitable” habitat within each MPA region is summarised in Table 6. The results (Tables 5 and 6) show that the West of Scotland (Territorial) MPA region is the most important region in terms of predicted habitat. The Net Gain, North Scotland (Territorial), South West Scotland (Territorial) and Northern Ireland are also important regions. Most significantly, the West of Scotland (Territorial) region loses 56% of its “most suitable” habitat by 2030 and 100% is lost by 2050.

**Table 5:** The area of “most suitable” (ms), “less suitable” (LS) and “unsuitable” (US) habitat within each MPA region.

**MPA Region**	**Area (Km^2^)**															
	**2009**			**2030**			**2050**			**2080**			**2100**			**Total area of region**
	**MS**	**LS**	**US**	**MS**	**LS**	**US**	**MS**	**LS**	**US**	**MS**	**LS**	**US**	**MS**	**LS**	**US**	
East Scotland	0	0	98761	0	0.00	99204	0	0	98961	0	0	99242	0	0	99210	101511
East Scotland (Territorial)	20	923	12391	11	817	12537	4.83	151	13167	0	0	13386	0	0	13380	13656
Balanced Seas	0	6	15013	0	0	15282	0	0	15294	0	0	15273	0	0	15202	17846
Finding Sanctuary	3	931	77999	0	2	79059	0	0	78739	0	0	79391	0	0	79028	95979
Irish Sea Conservation Zone	10	1493	15485	0	9	16687	0	0	16833	0	0	16730	0	0	16700	17551
Isle of Man	7	601	3159	0	6	3774	0	0	3780	0	0	3780	0	0	3780	4609
MCZ Project Wales	24	2283	13627	0	8	15917	0	0	15918	0	0	15951	0	0	15946	16375
Net Gain	583	7031	103228	81	3300	107776	6.50	213	111024	0	0	110931	0	0	111117	113204
North Scotland	0	0	22742	0	0	23247	0	0	23185	0	0	23130	0	0	23194	29967
North Scotland (Territorial)	324	1540	21361	136	1229	21662	32	232	22708	0	0	22996	0	0	23018	23860
Northern Ireland	210	683	3197	0	153	4090	0	21	4196	0	0	4160	0	0	4234	9071
South West Scotland (Territorial)	273	766	6538	37	258	7319	0	0	7598	0	0	7644	0	0	7645	7996
West Scotland	0	93	16698	0	0	17165	0	0	16650	0	0	17156	0	0	16942	27701
West Scotland (Territorial)	1345	2311	36913	590	1074	39231	0	382	40435	0	0	40698	0	0	40742	43640

**Table 6:** The area and percentage loss of “most suitable” habitat within each MPA region.

MPA Region	**2009**	**2030**		**2050**		**2080**		**2100**	
	**Area**	**Area**	**% Loss**	**Area**	**% Loss**	**Area**	**% Loss**	**Area**	**% Loss**
East Scotland	0.00	0.00	n/a	0.00	n/a	0.00	n/a	0.00	n/a
East Scotland (Territorial)	19.91	10.71	46	4.83	76	0.00	100	0.00	100
Balanced Seas	0.00	0.00	n/a	0.00	n/a	0.00	n/a	0.00	n/a
Finding Sanctuary	3.20	0.00	100	0.00	100	0.00	100	0.00	100
Irish Sea Conservation Zone	10.28	0.00	100	0.00	100	0.00	100	0.00	100
Isle of Man	7.29	0.00	100	0.00	100	0.00	100	0.00	100
MCZ Project Wales	24.11	0.00	100	0.00	100	0.00	100	0.00	100
Net Gain	582.81	80.86	86	6.50	99	0.00	100	0.00	100
North Scotland	0.00	0.00	n/a	0.00	n/a	0.00	n/a	0.00	n/a
North Scotland (Territorial)	323.64	136.15	58	31.72	90	0.00	100	0.00	100
Northern Ireland	210.26	0.00	100	0.00	100	0.00	100	0.00	100
South West Scotland (Territorial)	273.09	36.97	86	0.00	100	0.00	100	0.00	100
West Scotland	0.00	0.00	n/a	0.00	n/a	0.00	n/a	0.00	n/a
West Scotland (Territorial)	1345.32	590.39	56	0.00	100	0.00	100	0.00	100

## Discussion

The aim of this study was to model the ecological niche and bioclimatic envelope of *M* *. modiolus* beds within UK waters as a baseline for subsequent increased ocean temperature projections, and to demonstrate its application as a tool for future management of habitats. Species Distribution Modelling techniques have previously been applied in the marine environment to a range of motile species [28,29,32-35]; but, with the possible exception of Ross and Howell’s 2012 [9] study on deep sea organisms, this is the first study the authors are aware of that deals with marine habitat forming species of high conservation management interest, under an increasing ocean temperature scenario. In a terrestrial setting bioclimatic envelope models provide perhaps the best available guide for conservation managers and policy makers [2,4,36-39] and have been considered as first approximations of the magnitude and broad patterns of future impacts [2]. In this context, terrestrial conservation protection has appeared inadequate under future climate change scenarios [36]. For example, Carvalho et al. [36] concluded that protected areas covered 10% of the current distribution of all Iberian herptiles; and that to maintain this coverage the protected area network would have to be increased by 1-2% by 2080.

### Environmental Envelope Analysis

The Environmental Envelope Analysis (EEA) provided a relatively quick and simple method for analysing the potential distribution of the *M* *. modiolus* habitat and was performed in order to validate the Maxent model method. The EEA greatly improves the visualisation and analysis of potential projected conditions in support of conservation planning without the requirement for specialised modelling knowledge; and methods such as this demonstrate the possibilities of generating new knowledge from existing data sets. It was important that all environmental variable layers used were freely and publically available in order to demonstrate the immediate applicability of such modelling tools to inform contemporary policy and management decision making for the marine environment.

The envelope analysis, however, will only take into account areas where all the individual "preferred ranges" overlap, a concept that is corrected for within the Maxent model. In addition, the EEA does not lend itself sufficiently to model testing and statistical analysis, therefore it would not necessarily provide robust evidence, unless run alongside another model. It does however provide a robust representation of shifting habitats in a timely and cost-effective manner.

The EEA method developed within this study is, as far as the authors are aware, a new use of the method for the selection of an environmental envelope based on the interquartile range analysed within a GIS setting. Two other proposed methods were also investigated [40,41], but these methods were judged to be unsuitable for the data used within this particular study. These methods were either based on descriptive data and on species that inhabitat a very particular niche [41], or suggested too wide an envelope (minimum to maximum ranges) [40].

The envelope analysis utilised, predicts that the habitat will retreat northwards as sea temperature increase, with more limited extent of distribution in the Irish Sea and Shetland regions compared to the current known bed occurrence records (Figure 1). These results would suggest that although this type of analysis is useful for simple visualisation and summarisation of suitable habitat areas, more refinement of environmental layers is required for detailed application.

### Species Distribution Model

The Maxent model outputs in this study provide an overview of potentially suitable *M* *. modiolus* bed habitat. Despite the present model being built on environmental variables with coarse resolution, species with a narrow ecological niche can show high accuracy of predicted distribution under modelled conditions compared to those with a broader niche [29]. In addition, the global model which was utilised in this study closely resembled the output of the comparative environmental envelope analysis. Overall, therefore, the baseline trained model (global model) can be interpreted as a good predicted range, with projections showing that the *M* *. modiolus* beds lose their ability to fulfil that range by 2100. Under these modelled conditions *M* *. modiolus* beds in the UK will be increasingly vulnerable.

Details of climate change scenarios in the marine environment are poorly understood. The extent to which environmental changes (e.g. alterations to hydrodynamics and sediment dynamics) might occur alongside temperature increases is not well studied. Other environmental variables such as salinity and acidity were excluded in the present study because there was a lack of information [42], or conflicting literature on the potential levels and direction of change in these variables (e.g. salinity increasing [43,44], salinity decreasing [45]; salinity decreasing at high latitudes and increasing at low latitudes [46,47]). Under the climate change scenario A1B [25] ocean pH is predicted to decrease to 7.9 from a baseline of 8.1 in 2007. However, no environmental data on the variability of pH of the seawater around the UK was readily available to allow this scenario to be defined in terms of spatial variation. Depth was excluded from the "climate change" scenario based on the quality of the bathymetry data used. The sea level rise predicted under the climate change scenario A1B indicates an increase of up to 0.5 m by 2100.

The assumptions made on increased ocean temperature at depth in the present study are supported by research conducted by Levitus et al. [48]. This research suggested warming of the upper 300m of the world’s oceans between 1948 and 1998, particularly the Indian and Atlantic Oceans. However, it is unclear as to what magnitude ocean warming at depth will occur in the future; and variations in the speed of climate change between UK regions are unknown [49].

An issue with SDM techniques for sessile organisms like *M* *. modiolus* is that SDM, including Maxent, base predicted distributions on an ecological niche theory, and do not give consideration to propagule dispersal [50], dispersal vectors and propagule establishment [51]. Although knowledge of larval dispersal may not necessarily refine habitat suitability models in definite terms, it may lead to an enhanced understanding of model predictions or contribute to model accuracy.

Presently, little information is available on genetic connectivity of the beds. Holt et al. [18] and Comely [52] suggest recruitment from outside the area for beds off the Lleyn Peninsula and the Isle of Man; and self-sustaining populations occurring in Strangford Lough and the Scottish sealochs based on perceptions of wide dispersal from and to highly tidal areas, and low dispersal from and to sealochs with high water residence times.

*M* *. modiolus* are thought to spawn in a relatively narrow temperature window (7-10^o^C) [16] suggesting that, although the model shows a reduction of potentially suitable *M* *. modiolus* habitat, recruitment may be the mechanisms by which reefs cease to be viable. Established reefs may therefore persist beyond the prediction of the present study, but their reproduction may be hindered; and local adaption to the changing climate may occur over time.

*M* *. modiolus* are relatively long lived, with a life-span of approximately 20-100 years [53] giving some indication of the lag-time before senescence might be detected. There is, as yet, no evidence of reefs that are senescing. Many beds studied in the 1950s still exist [23,54-56] and reefs in North Wales are thought to have persisted for approximately 150 years [12], with evidence that these beds are still recruiting [56]. Studies have recorded an overall decline in the extent of *M* *. modiolus* beds in the period between 1950 and 1990 [53].

The trained model output illustrated that the most suitable baseline areas occurred in west Scotland, Northern Ireland (Strangford Lough) and Shetland, with less suitable habitat occurring in the Irish Sea and Orkney. Patches of suitability around the east coast of England (Norfolk coast) appear misleading because beds are not known to occur there (Figure 7). It is possible that the model is predicting the existence of suitable environmental conditions for *M* *. modiolus* beds in in this area, but other unaccounted factors (e.g. connectivity, fishing impacts, or turbidity etc.) could be preventing actual bed presence. Limitations of knowledge, low numbers of targeted surveys or decline of beds in this area are also possible explanations. For example, the Southern North Sea, the Western Channel/Celtic Sea and Irish Sea are known to have the highest intensity of trawling and dredging pressure in the UK [8]: an anthropogenic pressure thought to impact these biogenic habitats (e.g. Strain [57]). Furthermore, the North Norfolk Sandbanks and Saturn Reef are designated MPAs (Special Area of Conservation; SAC) for *Sabellaria* *spinulosa* Leuckart, 1849 beds, a tube dwelling polychaete, which require silty, turbid conditions to build their tubes and reefs [18]. In this study the model may therefore be interpreting the suitability of areas for biogenic reefs and may not be refined enough to distinguish the environmental envelope for functionally similar species structures. *S* *. spinulosa* requires suspended sediment to build their tubes, *M* *. modiolus* does not, and may be sensitive to smothering and/or lack of suitable suspended food.

### MPA Region Assessment

The area of the current SACs that encompass *M* *. modiolus* beds (Loch Creran and Lochs Duich, Long and Alsh beds, west Scotland; the Lleyn Peninsula and Sarnau, north Wales; Sanday, Orkney; Strangford Lough, Northern Ireland) cover 141Km^2^ of the predicted distribution of “most suitable” habitat in 2009; 15Km^2^ in 2030 and zero in 2050 to 2100. This represents 8% protection of the predicted “most suitable” habitat range in 2009 and this drops to 0.9% by 2030; and 0% by 2050. Protection is therefore limited, and will dwindle in contrast to the Convention on Biological Diversity target: “*By 2020, at least* ….. *10% of coastal and marine areas, especially areas of particular importance for biodiversity and ecosystem services, are conserved through … …*. *representative and well-connected systems of protected areas and other effective area-based conservation measures*….” Although, this statement is not species specific, the IUCN’s Vth World Parks Congress, 2003, suggested that 20-30% of each habitat should be protected within MPAs by 2012 [9,18,58-60].

Micheli et al. [61] concluded that the protection afforded to species in marine reserves supports population resistance to large scale environmental impacts. This is achieved through greater larval production and recruitment; large adult body size; absence of fishing related mortality and larval spill-over; maintained reproductive output; and recoverability. A network of marine protected areas may therefore be the most effective tool in mitigating the negative impacts of climate change on marine ecosystems and their associated livelihoods [61].

In addition to designated protected areas, consideration also needs to be given to potential dispersal corridors [37] to accommodate movement of conservation interest species/habitats within a changing climate, potentially safeguarding these areas through conservation easement [3].

### Pan-European perspectives

Presently, UK Good Environmental Status (GES) targets under the MSFD for rock and biogenic beds are drawn from the Habitats Directive [62] i.e. that the “Area is stable or increasing and not smaller than the baseline value” (EU Habitats & Species Directive, Council Directive 92/43EEC). This is in keeping with one of the key aims of the MSFD to “Protect and preserve the marine environment prevent its deterioration or, where practicable, restore marine ecosystems”. However, one of the key MSFD characteristics of Biodiversity (Descriptor 1) is that “The quality and occurrence of habitats and the distribution and abundance of species are in line with prevailing physiographic, geographic and climatic conditions”, a characteristic that is being interpreted as accommodating climate change [6,62]. The implication of the present study is that, in the short term, maintaining nationally “stable or increasing” areas of some protected habitats may not be achievable within the next 40 years without significant restorative and facilitated migration work. For habitats like these, the connectivity of an MPA network will be of paramount importance, especially for those that have already suffered historic loss and fragmentation. It is also possible that within a life time, maintaining “stable” areas may not be achievable at all within a national or regional context.

The amount of habitat loss that would be tolerated within the assessment of GES under the MSFD is yet to be defined for many target species/habitats and methods such as the one demonstrated within this study, could, with further refinement enable more plausible definition of targets.

## Conclusions

Paradoxically, the achievement of GES within ‘prevailing climatic conditions’ may require European Atlantic nations to manage the decline and migration of some of their marine habitats of biodiversity conservation importance rather than maintain their present extent. This concept is relatively novel to marine conservation management and not currently represented within national or international Marine Spatial Planning; nor in the conservation objectives or management plans of MPAs.
